# The New Proactive Approach and Precision Medicine in Crohn’s Disease

**DOI:** 10.3390/biomedicines8070193

**Published:** 2020-07-03

**Authors:** Eran Zittan, Ian M. Gralnek, Marc S. Berns

**Affiliations:** 1 Ellen and Pinchas Mamber Institute of Gastroenterology and Liver Diseases, IBD unit, Emek Medical Center, Afula 1834111, Israel; ian_gr@clalit.org.il; 2 Rappaport Faculty of Medicine Technion-Israel Institute of Technology, Haifa 31096, Israel; marcsberns@gmail.com

**Keywords:** IBD, Crohn’s disease, tight control, treat-to-target, objective measurement

## Abstract

The proactive approach to Crohn’s disease (CD) management advocates moving toward algorithmic tight-control scenarios that are designed for each CD phenotype to guide remission induction, maintenance therapy, active monitoring, and multidisciplinary care to manage the complexities of each inflammatory bowel disease (IBD) patient. This requires accurate initial clinical, laboratory, radiological, endoscopic, and/or tissue diagnosis for proper phenotypic stratification of each CD patient. A substantial proportion of patients in symptomatic remission have been reported to demonstrate evidence of active disease, with elevated fecal calprotectin(FC) and C-reactive protein (CRP) levels as a hallmark for mucosal inflammation. Active mucosal inflammation, and elevated CRP and fecal calprotectin (FC) have been shown to be good predictors of clinical relapse, disease progression, and complications in IBD patients. The next frontier of treatment is personalized medicine or precision medicine to help solve the problem of IBD heterogeneity and variable responses to treatment. Personalized medicine has the potential to increase the efficacy and/or reduce potential adverse effects of treatment for each CD phenotype. However, there is currently an unmet need for better elucidation of the inflammatory biopathways and genetic signatures of each IBD phenotype, so personalized medicine can specifically target the underlying cause of the disease and provide maximal efficacy to each patient.

## 1. Introduction

**Figure 1:**
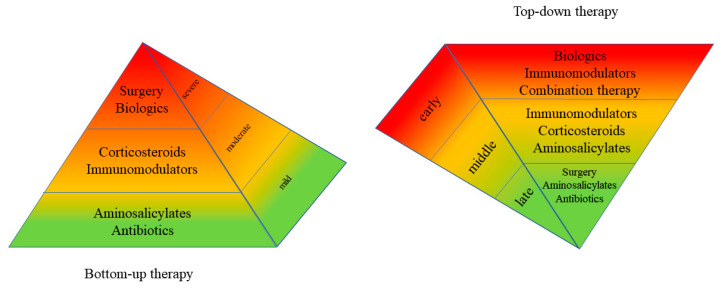
Bottom-up vs. top-down therapy.

Crohn’s disease (CD) is a chronic, immune-mediated inflammatory bowel disease (IBD) of unknown etiology marked by recurrent bouts of transmural inflammation that can affect the entire gastrointestinal tract (GIT) from the mouth to the anus, with the potential to cause significant physical and psychological morbidity [1]. The incidence of CD continues to rise in both the Western and developing worlds, imposing a heavy burden on healthcare systems across the globe [2,3]. While the rates of surgical intervention has been declining due to therapeutic advancements, approximately 70% of CD patients will ultimately require intestinal surgery at some point in their life [4,5]. Traditionally, CD has been treated with a “bottom-up” stepwise approach based on results from studies that used subjective, symptom-based disease activity indices like the Crohn’s disease activity index (CDAI) and the Harvey–Bradshaw index (HBI) to evaluate treatment efficacy [6]. This approach uses a stepwise intensification of treatment that initiates biologic therapy only after pharmacological therapies such as 5-aminosalicylic acid (5-ASA) medications, corticosteroids, and immunomodulators (purine analogs and methotrexate) have failed (Figure 1) [6,7,8]. The antithetical method, the “top-down” approach, of initiating remission induction with biologic therapy was later introduced to address the issue that significant numbers of patients were failing to respond to conventional pharmacological therapy [8]. Now, the “treat-to-target” or proactive approach is refining the goals of CD treatment by aiming to improve objective measurements of inflammation, including C-reactive protein (CRP) and fecal calprotectin (FC), in addition to patient-reported outcomes (PROs), using disease activity indices and biomarkers. This approach can enable physicians to make better-informed decisions to modify treatment as needed to achieve deep remission, i.e., clinical remission with mucosal healing [9]. The purpose of this review was to evaluate the body of literature on the proactive approach to managing CD and to make recommendations for the future of CD management. 

## 2. Natural History

The natural history of CD is marked by a variable disease course. Over a 10-year period, disease location tends to remain stable, while disease behavior tends to progress [10]. Therefore, a large proportion of CD patients develop penetrating and/or stricturing complications due to alternating periods of clinical remission and active disease flares of variable duration, which leads to cumulative intestinal damage [10,11,12]. This intestinal damage can be measured using the Lémann Index (LI), which combines cross-sectional imaging, with or without endoscopy depending on CD location, to objectively assess the severity of damage along the GIT at a single point in time [13]. Patients with progressive CD and increased intestinal damage, defined as a change between the first and last LI score greater than zero, are more likely to require surgery and utilize more healthcare resources [14]. 

Altering the natural history of CD to prevent disease progression and cumulative intestinal damage should be the long-term goal of therapy. Studies have demonstrated that patients with evidence of endoscopic mucosal healing within 1 year after the initial diagnosis of CD had lower rates of hospitalization, decreased risk for bowel resection, higher inflammatory bowel disease questionnaire quality of life (QoL) scores, and better long-term outcomes [15,16,17,18].

Anti-TNF-α therapy has demonstrated efficacy in altering the natural course of CD [19]. In addition, several studies which used the top-down approach such as, SONIC, and REACT studies have demonstrated either superior efficacy and/or safety of early combined immunosuppression compared to the standard step-up approach [20,21,22]. Therefore, it appears warranted to use biologic agents early in the disease course in order to aggressively combat TNF-α-mediated inflammation and to quickly achieve deep remission with mucosal healing. This more aggressive approach may help to minimize cumulative intestinal damage, hospitalization rates, surgery, incidence of neoplasm, costs of care, disease-related disability, and mortality by preventing complications from long-term corticosteroid exposure [20,22,23,24,25,26].

However, anti-TNF biologic agents are not appropriate for every CD phenotype. Dubinsky et al. formulated an anti-TNF non-response predictive model using genome-wide association studies to predict which patients would not benefit from anti-TNF therapy [27]. Alternative available biologic agents for use in CD unresponsive to anti-TNF include anti-IL-12/23 (ustekinumab), anti-α4 integrin (natalizumab), and anti-α4β7 integrin (vedolizumab) [28]. Biologic therapy has demonstrated efficacy in altering the natural course of CD [19]. Therefore, it is imperative to choose the management approach (biologics, immunomodulators, steroids and/or surgery) that best targets and addresses the underlying inflammatory mechanism(s) of the specific CD patient. 

## 3. Evidence for Top-Down Remission Induction in IBD

Inducing remission with a top-down approach of combined biologic and immunomodulator therapies may be more effective at inducing steroid-free remission in newly diagnosed CD patients than the conventional step-up approach, beginning with corticosteroids followed by sequential escalation to immunomodulators and anti-TNF. In the TOP-DOWN study, an open-label, multi-center, randomized trial, 60% (39/65) of patients achieved steroid-free remission in the early combined immunosuppression group (infliximab (IFX) combined with azathioprine (AZA)) compared with 35.9% (23/64) in the conventional treatment control group (bottom-up approach) at week 26 (*p* = 0.0062) [20]. Similarly, the steroid-free remission rates at week 52 remained significantly greater in the top-down group at 61.5% (40/65) compared with 42.2% (27/64) in the bottom-up group (*p* = 0.028).

Moreover, combined immunosuppression is more effective at inducing steroid-free remission than monotherapy using an anti-TNF or immunomodulator alone. The SONIC trial found that 56.8% (96/169) of patients on combination therapy (IFX and AZA) achieved steroid-free remission at week 26, compared to 44.4% (75/169) on IFX alone and only 30.0% (51/170) on AZA monotherapy [21]. Similarly, a meta-analysis of seven studies including 1984 patients assessed CD remission rates and found adalimumab (ADA) combination therapy to be significantly superior to ADA alone (OR = 0.78; *p* = 0.02) [29]. 

While the REACT study found no significant difference in rates of clinical remission between top-down early combined immunosuppression and bottom-up therapy after one year, there were significantly lower rates of complications and surgery in the top-down group after two years [22]. However, this studied used the subjective HBI rather than objective measures of disease activity to assess clinical remission rates, which may account for the inability of the study to detect differences in treatment. 

## 4. Proactive Optimization of CD Therapy with Tight Control

Treatment regimens for CD patients can be optimized with an active approach or tight-control scenario rather than a passive “wait and see” approach. A tight-control scenario is an algorithmic treatment pathway whereby physicians may modify treatment based on treat-to-target goals of patient-reported outcomes and/or laboratory data measured at predetermined intervals. For example, the CALM study was the first top-down study that compared remission rates of tight control versus conventional clinical monitoring to escalate or de-escalate biologic treatment [30]. At Weeks 1, 11, 23 and 35, the CDAI, CRP, and FC were used as targets of treatment in the tight-control group, while only the symptom-based CDAI was used in the conventional clinical monitoring group. After 48 weeks, the tight control group achieved significantly higher rates of deep CD remission with mucosal healing compared to the conventional treatment group based on the Crohn’s disease endoscopic index of severity (CDEIS), 46.0% (56/122) vs. 30.0% (37/122) (*p* = 0.01), respectively. 

Similarly, the TAXIT trial compared the efficacy of IFX escalation or de-escalation dosing to achieve remission after one year between a treat-to-target group of IFX trough concentration levels of 3–7 μg/mL versus conventional clinical monitoring (patient symptoms and CRP levels) [31]. While there were no statistically significant differences in the rates of CD remission after 1 year between the two groups (69.0% trough group vs. 66.0% clinical group; *p* = 0.69), the trough concentration dosing group had significantly fewer disease flares during treatment compared to the conventional clinical monitoring, 7.0% (9/128) vs. 17.0% (21/123) (*p* = 0.018), respectively. In addition, 92% (48/52) of CD subjects with IFX trough levels > 7 μg/mL were safely dose-de-escalated with a dose reduction of 5 mg/kg and infusion interval increase of 2 weeks, indicated by maintaining trough levels between 3–7 μg/mL, which led to 28% drug-cost savings (*p* < 0.001). 

Therapeutic drug monitoring of ADA in patients with both low trough levels and low titers of antibodies against ADA benefit from dose optimization and combined immunosuppression [32,33]. One study demonstrated that mucosal healing in CD patients was strongly associated with higher trough concentration levels of ADA (median = 14.7 μg/mL) compared to non-mucosal healing patients who had lower trough levels (median = 3.4 μg/m; *p* = 0.00006) [34]. The same study found the optimal ADA trough concentration cutoff for endoscopic mucosal healing to be 8.1 μg/mL, which resulted in 91.4% sensitivity, 76.0% specificity, 84.2% positive predictive value (PPV), and 86.4% negative predictive value (NPV), suggesting that physicians can better manage CD patients on ADA by targeting a specific trough level cutoff to better achieve mucosal healing. 

In the POCER trial, a proactive approach to therapy was found to be more effective at preventing post-surgical endoscopic disease recurrence at 18 months compared to standard care [5]. The proactive approach in this study included colonoscopy at 6 months post-surgery, in addition to CDAI assessment, to examine the anastomosis site for recurrent inflammation based on the Rutgeert’s score (≥i2), with subsequent treatment escalation if needed. The standard care group did not receive post-surgical colonoscopy; hence, their disease was only monitored using the symptom-based CDAI, and CD treatment modifications were made based on patient symptoms. A proactive approach to CD disease monitoring and management via tight control appears to provide superior patient outcomes, because it arms the physician with objective clinical data to make better informed therapeutic decisions, as demonstrated in the prospective trials (Table 1). 

## 5. Objective Measurements of CD Activity

There is a disconnect between the clinical symptoms of CD and mucosal inflammation [35,36]. Patients presenting with severe clinical symptoms, for example abdominal pain, altered bowel movements, nausea, and vomiting, may have little objective evidence of mucosal inflammation and thus be over-treated. On the other hand, those with significant mucosal inflammation may be under-treated due to mild or absent symptoms and may not seek medical attention. Thus, patient-reported symptoms alone are not a reliable guide for the clinician in adjusting CD treatment regimens to control inflammation. While endoscopy is the diagnostic gold standard in IBD and has demonstrated its ability to be a useful tool in the active monitoring of the disease, it is time-consuming for the physician to perform and its invasive nature presents finite risks, inconvenience, and costs to the patient. In addition, standard ileocolonoscopy is unable to assess the small intestine proximal to the terminal ileum. Thus, there exists a need for more specific and reliable IBD biomarkers that can bridge the gap between subjective symptoms and objective endoscopic evidence to provide the physician with reliable data about the degree of intestinal mucosal inflammation in CD.

IBD patients with active inflammation have been shown to have significantly increased levels of FC, lactoferrin, polymorphonuclear neutrophil elastase (PMN-e), and serum CRP compared to those in a quiescent disease state [37]. Fecal measurements (e.g., FC, lactoferrin, and PMN-e) have been shown to be more specific and have superior predictive accuracy of endoscopic disease activity compared to serum biomarkers [37,38,39,40,41]. Specifically, FC correlates more strongly with the simple endoscopic score for Crohn’s disease (SES-CD) than serum CRP (Table 2) [42]. Although FC and CRP measurements are becoming more widely adopted in IBD clinics, they remain non-specific biomarkers of inflammation. Novel biomarkers like the molecular chaperone heat shock protein-60 (Hsp60) are currently in the experimental phase as potential new indicators of IBD activity.

### 5.1. Fecal Calprotectin

FC is a biochemical assay that measures calprotectin, a 36 kDa granulocyte protein of the S100 family with antimicrobial properties that binds zinc and calcium in stool samples [59]. FC levels correlate strongly with clinical and mucosal disease activity in colonic CD and UC [47,48]. A study by Zittan et al. demonstrated that an FC cutoff of >100 μg/g was 92% sensitive and 56% specific in predicting clinical disease activity, and 100% sensitive and 64% specific in predicting endoscopic disease activity; a cutoff of >200 μg/g was 81% sensitive, 53% specific, and 86% sensitive, 93% specific for clinical and endoscopic disease activity, respectively [48]. This study also demonstrated that low FC of <100 μg/g had the highest sensitivity and strongest negative predictive value in assessing clinical and endoscopic remission, and was strongly correlated with histological remission and the absence of basal plasmacytosis in colonic CD.

The correlation between FC and small-bowel CD is less straightforward. One study claimed that FC was equally sensitive in both small-bowel and colonic CD [60]. Meanwhile, another showed that FC correlated well with the degree of ileal inflammation on cross-sectional imaging and surgical pathology [61]. However, more recent studies have challenged the efficacy of FC as a surrogate marker for small-bowel CD activity [47,48,62,63,64]. One study found poor correlation between FC and endoscopic disease activity in isolated small-bowel CD [47]. A cutoff value of >100 μg/g had a sensitivity of 75% and a specificity of 50%, in addition to a low NPV of 50%. Conversely, this same study demonstrated a strong correlation with colonic CD and endoscopic disease activity, with a sensitivity of 100%, a specificity of 67%, and NPV of 100% at the same FC cutoff value. Another study found that FC levels may not be markedly elevated even in the presence of large ileal ulcerations [63]. Thus, FC cannot be recommended for use in diagnosing or monitoring disease activity in the small intestine, although it remains an excellent biomarker for colonic inflammation in CD. However, stool collection can be burdensome and uncomfortable for many patients, which may lead to poor adherence, as evidenced by a study evaluating FC test compliance in a CD cohort where only 35% (37/101) of participants performed the test [64].

### 5.2. CRP

CRP is an acute-phase reactant produced by hepatocytes and is a non-specific marker of inflammation. It is upregulated in response to inflammation and functions as an opsonin to promote phagocytosis. CRP is easily measured in the serum of routine blood tests. Its effectiveness as a predictor of disease activity in CD may be related to the location of active inflammation, as CRP was found to be most strongly correlated with CDAI scores in the disease of the colon compared to the small bowel [50]. A meta-analysis of 19 studies (*n* = 2499 IBD patients) assessing the relationship between CRP and endoscopic disease activity found a pooled sensitivity of 49% and specificity of 92% with CRP cutoffs of ≥5, 7, and 10 mg/L [39]. Another study found a statistically significant relationship between elevated CRP and endoscopic activity (*p* = 0.001) [65]. The main limitation of CRP is that it is non-specific to intestinal inflammation and can be elevated due to infection or extraintestinal inflammation. In addition, CRP levels are not elevated in 20–25% of relapsing CD patients due to single-nucleotide polymorphisms affecting the *CRP* gene [36].

### 5.3. Hsp60

Hsp60 is a chaperonin, a component of the molecular chaperone system, that helps proteins fold correctly and protects against cellular stress by preventing protein misfolding, premature degradation, or inappropriate aggregation [65]. Chaperonins have a significant role in activating the immune system, leading to inflammation [43,66]. Levels of Hsp60 increase substantially in the cytosol in response to cellular stress and are excreted extracellularly. Upregulation of Hsp60 has been demonstrated to be part of the extreme inflammatory response of several pathologies, including IBD, where it was found in abundance in the cytoplasm of epithelial cells in both CD and UC during active disease [67]. While this study demonstrated significantly increased cytosolic Hsp60 levels in active IBD versus healthy controls, this study was performed using immunohistochemistry, immunofluorescence, and Western blotting from tissue samples, which may only be obtained via invasive endoscopy or surgery and thus are not suitable as a source of routine biomarkers. However, a recent study from 2019 demonstrated that serum Hsp60 was significantly higher in patients with colorectal cancer compared to controls [68]. To our knowledge, a similar study has not yet been performed in any IBD cohort to demonstrate increased serum Hsp60 levels in patients with active inflammatory IBD versus healthy controls. Further clinical research is needed to determine whether serum Hsp60 could be the biomarker of the future.

### 5.4. Multifactorial Disease Activity Indices

As previously mentioned, symptom-based disease indices are poor predictors of endoscopic severity. Subjective disease activity indices like the CDAI and HBI correlate poorly with objective endoscopic findings [69,70]. Thus, augmenting disease activity indices with laboratory biomarkers into a single index is a sensible method to potentially improve the diagnostic predictability of actual endoscopic disease activity in CD (Table 2). The HBI-PRO is one such tool that combines the HBI with modified patient-reported outcomes (PROp), clinician-reported outcomes (PROc), and CRP [45]. In a study by Zittan et al., the HBI-PRO was found to be both more sensitive and specific to endoscopic disease activity than the HBI or CRP alone. Another tool is the PRO+, which combines patient-reported outcomes (PRO) and FC. The PRO+ was shown to significantly improve the specificity for predicting SES-CD endoscopic disease activity compared to a PRO without FC (88% vs. 55%, respectively) [46]. The development of more objective indices such as the HBI-PRO and PRO+ is an exciting endeavor that has the potential to enhance the physician’s ability to proactively monitor disease activity and make better-informed decisions. However, validation of these instruments in future studies is required before they can be recommended for routine use. 

### 5.5. Small-Bowel Cross-Sectional Assessment

Cross-sectional imaging modalities such as computed tomography (CT) and magnetic resonance imaging (MRI) can provide information in IBD about the extent and distribution of intraluminal disease and extraluminal complications, especially in the small bowel, which is not easily accessed by traditional endoscopy [56]. CT enterography (CTE) and MR enterography (MRE) detect abnormalities of the small-bowel mucosa with high accuracy via contrast-enhanced, thin-sliced imaging [56]. MRE may be a better imaging modality for the patient, as it avoids the ionizing-radiation exposure of CTE while providing similar or improved diagnostic accuracy; however, the cost is still prohibitive for many patients and/or institutions [57,58]. Moreover, MRE is a critical tool to diagnose and characterize fistulizing CD, with a diagnostic specificity between 76–100% [51]. It can visualize fistula anatomy, help plan or monitor treatment, and rule out complications such as abscesses [71].

A more recent diagnostic advancement in evaluating the small bowel is video capsule endoscopy (VCE) [52,53]. In addition to its superior detection rates of proximal small-bowel lesions, VCE has been shown to have a higher sensitivity and specificity in detecting CD lesions of the terminal ileum (100% and 91%, respectively) compared to MRE (81% and 86%, respectively) and CTE (76% and 85%, respectively) [54]. However, an important complication of VCE is capsule retention, which can be caused by strictures or stenosis, adhesions, erosions, intestinal neoplasm, mesenteric ischemia, external compression, or other factors [55]. A systematic review of 11 studies in patients with established IBD found a pooled video capsule retention rate of 8.2% (95% CI = 6–11%; *p* = 0.000), and required removal via surgical resection or endoscopic video capsule removal in most cases [55].

CTE, MRE, and CE provide valuable objective data that may alleviate the guesswork of interpreting laboratory values in the assessment of CD inflammation and disease activity, especially in the small intestine, which is mostly inaccessible to the more invasive traditional endoscopic procedures (Table 2). They are the mainstay of small-bowel CD diagnosis and monitoring, and should be incorporated into any proactive approach to managing CD.

## 6. Multidisciplinary Care

The proactive approach to IBD management requires not only proactive disease monitoring and evidence-based therapy using objective targets, but also the management of other medical and psychological comorbidities, and the optimization of dietary and nutritional needs. This requires a multidisciplinary team approach facilitated by a shared electronic medical record that includes the primary care physician, other medical specialists including psychology/psychiatry as needed, and a dietician in addition to the IBD specialist.

It has been proposed that psychosocial care for IBD patients can reduce barriers to treatment, increase adherence to treatment plans, and provide better patient outcomes [72]. For example, psychological sequelae including anxiety and depression are common in IBD patients. Studies have found that psychological comorbidities are associated with decreased medication compliance and higher rates of relapse and hospitalization [73,74,75]. Treatment of comorbid psychological disorders in IBD is beneficial for improving patient quality of life and potentially reducing stress-induced disease exacerbation [1]. One study found that 6 months of antidepressant therapy was associated with decreased anxiety and depression, and improved disease activity (decreased CDAI), QoL, and sexual functioning [76]. Cognitive behavioral therapy (CBT) has also been shown to improve QoL, but may not have an effect on disease activity [77].

As for nutrition and diet, a recent Cochrane review that included 18 randomized controlled trials (*n* = 1878) was unable to draw any conclusions or make recommendations with regard to the efficacy of specific dietary interventions on remission induction and maintenance of IBD [78]. 

However, recent data of the Crohn’s disease exclusion diet (CDED) with or without partial enteral nutrition (PEN), has shown positive results for the remission induction of mild to moderate Crohn’s disease, especially in the pediatric population [79,80,81]. The 2019 study by Levine et al. using this dietary strategy was able to achieve a corticosteroid-free remission rate of 75% (30/45) by Week 6 and 75.6% (28/37) by Week 12 in a pediatric cohort [79]. This study compared CDED plus PEN to exclusive enteral nutrition (EEN), and the efficacy of CDED plus PEN to induce corticosteroid-free remission reached statistical significance over EEN after 12 weeks of therapy. Similarly, a 2017 CDED plus PEN study by Sigall-Boneh et al. evaluated a mixed cohort (*n* = 47) of 13 adults and 34 children in which remission was obtained in 69% (9/13) and 70% (24/34), respectively [81]. 

Disease-modifying diets aside, IBD patients may have reduced dietary intake due to poor appetite or nausea, and/or malabsorption. This may lead to nutrient deficiencies that require tailored diets and interventions to meet the specific nutritional needs of each patient in order to maintain a healthy body mass index (BMI) and to prevent nutrition-related complications, such as B12 deficiency following ileocecal resection [82].

Probiotic dietary supplements have been a widely debated topic in the realm of IBD. In recent years, the knowledge regarding the immunoregulatory effects of gut microbiota and its possible role in the pathogenesis of IBD has expanded [83,84]. Thus, probiotics have been proposed to alter the gut microbiota with the goal of decreasing stimulation of the intestinal immune system, preventing epithelial dysfunction, and reducing permeability of the gut mucosa. A long-term UC study comparing daily oral mesalazine 1200 mg monotherapy to daily oral mesalazine 1200 mg in combination with bidaily probiotics (blend of *Lactobacillus salivarius*, *Lactobacillus acidophilus*, and *Bifidobacterium bifidus* strain BGN4) found significant disease improvement based on the Modified Mayo Disease Activity Index (MMDAI) after 18 months of treatment with combination therapy compared to monotherapy [85]. Other studies found that probiotics, specifically VSL#3, were as effective as mesalazine in preventing UC relapse and were an effective treatment for pouchitis [86,87]. While the data on probiotics are generally positive in the UC cohort, the opposite seems to be true in the CD cohort, as the efficacy of probiotics remains unconvincing [88]. Probiotics are generally well-tolerated and have been used safely in patients for years; therefore, we recommend adjuvant probiotic therapy for UC [89]. In short, IBD patients are individuals that have unique dietary, nutritional, and psychosocial needs that could be optimized by a multidisciplinary team.

## 7. A Proactive Algorithmic Approach

**Figure 2:**
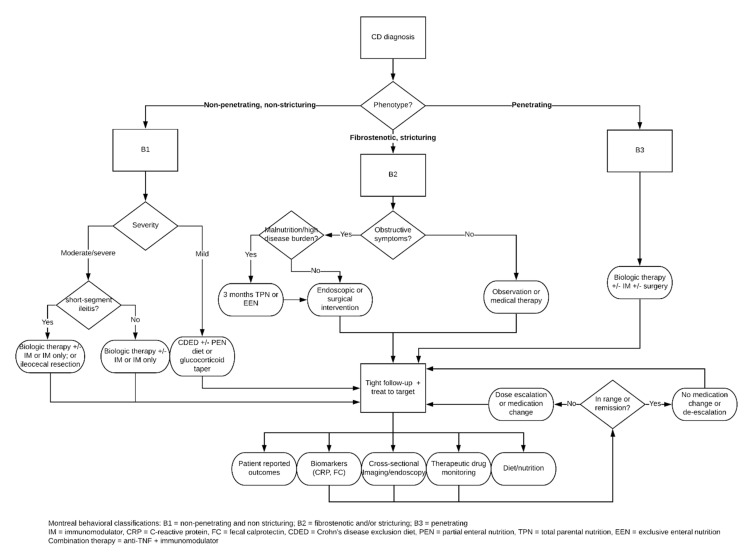
The new proactive algorithm.

The Montreal classification system classifies CD phenotypes based on age, disease location, and behavior, which can help to direct treatment [28,90]. We devised an algorithmic approach to CD management based on the Montreal behavior (B) subphenotypes: B1—non-penetrating and non-stricturing disease; B2—fibrostenotic and stricturing disease; and B3—penetrating disease (Figure 2). The patient at the time of diagnosis is first classified as either B1, B2, or B3 based on their initial presentation and full work-up. 

B1 phenotypes with mild disease activity tend to respond well to remission induction with glucocorticoids: budesonide for ileal and/or proximal colonic CD or prednisone for distal colonic involvement [28,91,92]. No clinical trials to date have evaluated various corticosteroid-tapering regimens in CD, so the length of taper is based on the clinical judgement of the physician. However, a standard tapering strategy is recommended to quickly identify patients who are not responding corticosteroid treatment and require additional therapy with immunomodulators and/or anti-TNFs. It is recommended that all patients taking corticosteroids also receive osteoprotective therapy with calcium and vitamin D supplements [93,94]. 

A possible alternative remission-induction strategy to corticosteroids for B1 mild ileal CD that may be considered is the CDED with or without PEN dietary intervention, as mentioned above. Data from several studies in recent years are positive and have shown relatively high corticosteroid-free remission-induction rates [79,80,81]. This strategy may help clinicians to reduce patients’ exposure to the deleterious effects of glucocorticoids. Despite these promising results, this remission-induction strategy is in its infancy, and strong clinical judgement needs to be exercised when considering this intervention for a patient. 

Maintenance options following successful tapering include observation with colonoscopy after 6 months, low-dose corticosteroids, immunomodulators, and/or anti-TNFs [92]. Observation alone is not recommended at this time due to the lack of evidence of long-term efficacy, and long-term corticosteroid use is not advised due its well-established side-effect profile. Therefore, maintenance therapy for even mild, non-penetrating, non-stricturing disease should consist of immunomodulators for localized disease and biologics with or without immunomodulators in extensive disease [91,92]. In general, immunomodulators and/or biologics in the B1 cohort are indicated for no response to initial corticosteroid treatment, steroid-dependence after failure to taper, moderate/severe disease, and maintenance therapy [28,91,92]. 

Furthermore, recent studies have demonstrated similar efficacy between conventional long-term drug therapy and early ileocecal resection for short-segment ileitis in patients with non-stricturing CD [95,96]. Early short-segment resection may be a suitable alternative for patients with contraindications to medication or preference to avoid long-term drug therapy and the associated side effects.

Management of the B2 fibrostenotic phenotype with obstructive symptoms due to fibrosis lies in the domain of surgery and endoscopy. In the case of obstructive ileocecal CD with minimal active inflammation, surgery is recommended [93]. Strictureplasty is a safe and effective alternative to resection of the small intestine and is generally recommended for stricture lengths <10 cm; however, some data exist to support the use of this procedure on longer segments with positive results [97,98]. Alternatively, endoscopic balloon dilation (EBD) is a first-line bowel-conserving procedure for the treatment of strictures and short-segment stenosis that has a favorable safety, efficacy, and patient satisfaction profile [99,100].

Treatment for the B2 phenotype without obstructive symptoms is controversial. There are neither any effective anti-fibrotic agents in existence, nor anti-inflammatory or immunosuppressive therapy that have been proven to prevent stricture formation [101,102]. Even inflammatory strictures are likely to have some degree of fibrosis, making medical treatment challenging [102,103]. Although the efficacy of anti-inflammatory and immunosuppressive therapy is limited in the fibrostenotic phenotype, current data support combination therapy with an anti-TNF and immunomodulator as the best long-term treatment option to prevent therapeutic failure resulting in endoscopic or surgical intervention due to obstructive symptoms secondary to stricturing and/or stenosing [104,105,106]. Observation without medical therapy may be appropriate in the case of a small focal stricture without evidence of inflammation.

There is evidence to suggest that B2 fibrostenotic patients with obstructive symptoms exhibiting signs of malnutrition and/or radiologic and/or endoscopic evidence of high disease burden benefit from a 3 month preoperative course of total parental nutrition (TPN) or exclusive enteral nutrition (EEN) to help improve both malnutrition and disease burden [107]. This may result in improved post-surgical outcomes due to decreased complications such as infection or anastomotic leak and/or less extensive resection as a result of the decreased disease burden. Furthermore, patients who respond exceptionally well to the TPN or EEN regimen and achieve symptom-free remission may be able to avoid surgery altogether [107].

B3 patients with penetrating disease should be aggressively treated with biologic monotherapy, or in combination with immunosuppressants and surgery if needed. Although anti-TNF monotherapy is effective at maintaining anti-TNF induced remission, it is strongly recommended to add an immunomodulator due to the potential for immunogenicity and loss of response [28]. Patients who enter remission with combination therapy with a biologic and immunomodulator should remain on the same regimen for maintenance therapy [91]. Current data support the notion that combination therapy is more effective than either anti-TNFs alone or immunomodulators alone [21,29]. Purine analogs are the preferred immunomodulators for combined immunosuppression; however, methotrexate can be used in patients who do not tolerate purine analogs [92]. Before initiating anti-TNF therapy, patients must be screened for latent and active tuberculosis and other latent opportunistic infections to avoid reactivation and secondary infections [28,91]. Alternative biologics to the anti-TNF agents include vedolizumab (anti-α4β7 integrin), natalizumab (anti-α4 integrin), and ustekinumab (anti-IL-12/23). Patients must test negative for anti-JC virus antibodies before starting natalizumab and continue JC virus antibody testing at least every 6 months thereafter due to the associated risk of developing progressive multifocal leukoencephalopathy (PML) [28,108,109]. We recommend reserving natalizumab as a last-line therapeutic agent due to the risk of PML.

In our algorithm, all management approaches converge to a treat-to-target follow-up protocol, which evaluates patients’ disease activity and response to treatment based on clinical and objective (laboratory/endoscopic) parameters. Examples of treat-to-target in CD include achieving mucosal healing based on endoscopic evaluation, FC levels <100 μg/g, or IFX trough concentration levels between 3–7 μg/mL. In addition to aiming for objective targets, the follow-up should include subjective patient-reported outcomes, objective laboratory (CRP and FC) and/or cross-section imaging (MRE and CTE), and/or upper and lower GI endoscopic data, assessment of dietary and nutritional needs, and evaluation of other medical/psychiatric comorbidities to obtain a complete picture of each patient’s health and well-being. Moreover, patients on biologic and immunosuppressant therapy may benefit from therapeutic drug monitoring to aid in drug optimization and prevent toxicity by measuring trough levels and assessing for the development of anti-drug antibodies [110,111,112,113,114].

Follow-up intervals are variable and should be tailored to suit the individual needs of each patient based on the clinical judgement of the physician. However, the follow-up time generally ranges from 2 weeks to 6 months, depending on a variety of factors such as medication class, response to treatment, disease severity, and individual historical trends. For example, follow-up during remission induction with corticosteroids is recommended within 2 weeks to monitor the initial response and to adjust or maintain the treatment plan accordingly [115]. In general, clinical and laboratory monitoring to initial CD treatment or remission induction should occur within 3 months and endoscopic or transmural evaluation within 6 months [116]. 

In addition to using trough levels as a target of treatment, therapeutic drug monitoring is recommended after remission-induction failure of or loss of response to immunomodulator and/or biologic therapy, with adjustment as follows: (1) increase the drug dose if both drug concentration and anti-drug antibodies are undetectable or low; (2) switch drugs within the same class if drug concentration is undetectable or low and anti-drug antibodies are high; and (3) if drug concentration is in the therapeutic range and anti-drug antibodies are undetectable or low, then switch drug classes if inflammation is present or continue drug regimen and look for other causes of symptoms if inflammation is not present [92]. Endoscopic or cross-sectional evaluation of the bowel is recommended in cases of relapse, refractory disease activity, and new unexplained symptoms [116]. Furthermore, extramural complications such as an abscess or fistula should be evaluated with cross-sectional imaging in addition to clinical and laboratory assessments to better characterize the lesion(s) and aid in developing the therapeutic plan [116]. Together, these data equip physicians with the knowledge to make informed decisions to best help their patients in both the short and long term.

## 8. Discussion

The proactive approach to CD management advocates moving toward algorithmic tight-control scenarios that are designed for each CD phenotype to guide remission induction, maintenance therapy, active monitoring, and multidisciplinary care to manage the complexities of each IBD patient. This requires accurate initial radiological, endoscopic and/or tissue diagnosis for proper phenotypic stratification according to the Montreal classification. 

A key aspect of this approach is therapeutic drug monitoring, which moves us one step closer to personalized medicine by enabling physicians to “treat-to-target” for each patient by adjusting medication doses and switching drug classes based on trough levels and anti-drug antibodies. Preliminary data suggest that high post-induction anti-TNF levels may help to predict clinical and laboratory remission, while the presence of anti-TNF antibodies and low anti-TNF levels may predict primary and secondary loss of response, respectively [117]. Thus, incorporating therapeutic drug monitoring into the standard of care enables IBD clinicians to be proactive regarding dose escalation and drug selection to aggressively combat inflammation with the goal of improving short- and long-term outcomes for patients. 

Currently, however, the treatment protocols of the B1, B2, and B3 CD phenotypes do not differ significantly, as the conventional evidence-based treatment algorithms have been designed based on disease severity. There is a need for new evidence-based treatment strategies that factor in disease behavior in addition to severity. For example, fibrostenotic disease behavior (B2) has traditionally been managed with anti-inflammatory and immunosuppressive therapy in addition to surgery as needed. However, early immunosuppression in high-risk patients has not been shown to reduce the frequency of stricturing [101]. In fact, corticosteroids have been linked to an increase in procollagen gene expression, synthesis and secretion by smooth muscle cells in the human GIT, and failure of long-term fibrostenotic therapy [104,118]. The efficacy of anti-TNF monotherapy in stricturing CD is also controversial. Some early data suggested an increased rate of intestinal obstruction with IFX monotherapy [118,119,120,121]. Meanwhile, other studies have argued the opposite [23,122,123,124]. To date, the most effective medical therapy in high-risk patients with moderate to severe CD seems to be combination anti-TNF and immunomodulator therapy, while endoscopic and surgical interventions remain important options as needed [104,105,106]. However, the current medical therapy against fibrostenotic CD is suboptimal, and new effective strategies that target the mechanisms of fibrosis are needed to prevent more invasive procedures. 

A new possible alternative remission-induction strategy to corticosteroids for B1 mild ileal CD that may be considered is the CDED with or without PEN dietary intervention, as mentioned above. Data from several studies in recent years are positive and have shown relatively high corticosteroid-free remission-induction rates [79,80,81]. This strategy may help clinicians to reduce patients’ exposure to the deleterious effects of glucocorticoids. Despite these promising results, this remission-induction strategy is in its infancy, and strong clinical judgement needs to be exercised when considering this intervention for a patient. 

In the interim, there is renewed interest in pre-operative nutrition optimization in B2 phenotypes with obstructive symptoms. Studies are currently evaluating the efficacy of TPN or EEN in malnourished B2 patients with respect to pre- and post-surgical outcomes [107]. If patients are malnourished, defined as albumin <3.5 g/dL, significant weight loss, and cachexia, then 1–3 months of EEN or TPN may improve surgical outcomes [107].

The next frontier of treatment is personalized medicine or precision medicine to help solve the problem of IBD heterogeneity and variable responses to treatment [125]. Personalized medicine has the potential to increase the efficacy and/or reduce potential adverse effects of treatment for each CD phenotype. However, there is currently an unmet need for better elucidation of the inflammatory biopathways and genetic signatures of each IBD phenotype, so personalized medicine can specifically target the underlying cause of the disease and provide maximal efficacy to each patient.

## Figures and Tables

**Table 1:** Evidence for the proactive management of Crohn’s disease.

Study	Country	Design	Cohort	Total Patients	Treat-to-Target Protocol	Primary Endpoint	Results
CALM	Multiple	prospective RCT	Adults with active endoscopic CD	244	Clinically based (standard care) vs. tight control (proactive care) ADA dosing based on FC, CRP, and CDAI	Mucosal healing (CDEIS < 4) without deep ulcers at 48 weeks	46% proactive care vs. 30% standard care
TAXIT	Belgium	prospective RCT	Adults with moderate-to-severe CD or UC	263 (CD = 178, UC = 85)	Clinically based (standard care) vs. concentration-based IFX (proactive care) dosing	Clinical remission at 1 year	62.6% proactive care vs. 54.9% standard care (CD)
POCER	Australia & New Zealand	prospective RCT	Adults with CD undergoing intestinal resection	184	Clinically based (standard care) vs. endoscopic disease monitoring (proactive care)	Post-surgical endoscopic remission at 18 months	51% proactive care vs. 33% standard care

ADA = adalimumab; FC = fecal calprotectin, CRP = C-reactive protein, CDAI = Crohn’s disease activity index, IFX = infliximab, CDEIS = Crohn’s disease endoscopic index of severity.

**Table 2:** Predictors of endoscopic disease activity.

Assessment	Cutoff Value	Sensitivity	Specificity	References
*Questionnaires*				
CDAI	≥150	24–38%	72–100%	[9,37,40,43]
HBI	>4	57%	76%	[40,44,45]
PRO		61%	55%	[45,46]
PRO+		63%	88%	[45,46]
*Biomarkers*				
FC	>100 μg/g	67–100%	57–64%	[35,36,37,38,39,40,42,44,47,48]
FC	>200 μg/g	47–86%	78–93%	[35,36,37,38,39,40,42,44,47,48]
FC	Pooled	88%	73%	[35,36,37,38,39,40,42,44,47,48]
CRP	≥5 mg/L	24–70%	64–100%	[36,37,38,39,41,42,44,49,50]
CRP	Pooled	49%	92%	[37,39,41,44,49,50]
Lactoferrin	>0.725	85%	77%	[37,39,40,41]
Lactoferrin	Pooled	82%	79%	[37,39,40,41]
*Imaging*				
VCE		100%	91%	[51,52,53,54,55]
MRE		81%	86%	[51,54,56,57,58]
CTE		76%	85%	[51,54,57]

FC = fecal calprotectin; CRP = C-reactive protein; PMN-e = polymorphonuclear leukocyte elastase; CDAI = Crohn’s disease activity index; HBI = Harvey–Bradshaw Index; PRO = patient-reported outcomes; PRO+ = patient-reported outcomes + biomarkers; VCE = video capsule endoscopy; MRE = magnetic resonance enterography; CTE = computed tomography enterography.
